# 2-(Dimethyl­amino­meth­yl)phenyl phenyl telluride

**DOI:** 10.1107/S1600536809038161

**Published:** 2009-09-26

**Authors:** Tapash Chakraborty, Harkesh B. Singh, Ray J. Butcher

**Affiliations:** a Department of Chemistry, Indian Institute of Technology Bombay, Powai 400 076, India; b Department of Chemistry, Howard University, 525 College Street NW, Washington, DC 20059, USA

## Abstract

The title compound, C_15_H_17_NTe, is a heteroleptic Te, *N*-bidentate ligand having a short Te⋯N contact [2.8079 (16) Å] involving a secondary bonding inter­action between the amino N and Te^II^ atoms. The Te—C bond [2.136 (2) Å] *trans* to the amino group is slightly elongated compared to the other Te—C bond [2.1242 (18) Å] due to the hypervalent inter­action. The bond angle for the *trans* N—Te—C atoms [164.92 (6)°] deviates significantly from linearity.

## Related literature

For Heck and cross-coupling reactions, see: Cella *et al.* (2006 ▶); Nishibayashi *et al.* (1996*a* ▶,*b* ▶); Zeni & Comasseto (1999 ▶); Zeni *et al.* (2001 ▶). For intra­molecularly coordinated tellurides, see: Detty *et al.* (1995 ▶); Drake *et al.* (2001 ▶); Engman *et al.* (2004 ▶); Kaur *et al.* (1995 ▶, 2009 ▶); Menon *et al.* (1996 ▶); Panda *et al.* (1999 ▶); Singh *et al.* (1990 ▶). For van der Waals and covalent radii, see: Bondi (1964 ▶); Cordero *et al.* (2008 ▶).
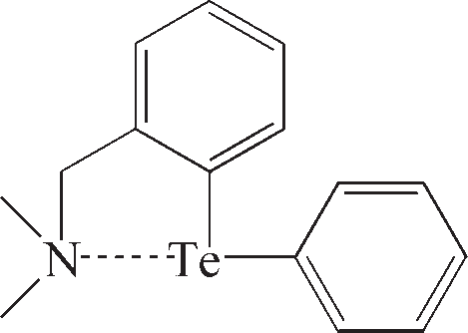


## Experimental

### 

#### Crystal data

C_15_H_17_NTe
*M* *_r_* = 338.90
Monoclinic, 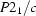

*a* = 8.5736 (3) Å
*b* = 13.2472 (5) Å
*c* = 12.6719 (4) Å
β = 95.933 (3)°
*V* = 1431.52 (9) Å^3^
*Z* = 4
Mo *K*α radiation
μ = 2.06 mm^−1^
*T* = 110 K
0.49 × 0.41 × 0.27 mm

#### Data collection

Oxford Diffraction Gemini R CCD diffractometer
Absorption correction: multi-scan (CrysAlis Pro; Oxford Diffraction, 2009 ▶) *T*_min_ = 0.728, *T*_max_ = 1.000
20642 measured reflections
4836 independent reflections
2926 reflections with *I* > 2σ(*I*)
*R*_int_ = 0.028

#### Refinement

*R*[*F* ^2^ > 2σ(*F* ^2^)] = 0.023
*wR*(*F* ^2^) = 0.058
*S* = 0.97
4836 reflections
156 parameters
H-atom parameters constrained
Δρ_max_ = 0.58 e Å^−3^
Δρ_min_ = −0.47 e Å^−3^

### 

Data collection: *CrysAlis Pro* (Oxford Diffraction, 2009 ▶); cell refinement: *CrysAlis Pro*; data reduction: *CrysAlis Pro*; program(s) used to solve structure: *SHELXS97* (Sheldrick, 2008 ▶); program(s) used to refine structure: *SHELXL97* (Sheldrick, 2008 ▶); molecular graphics: *SHELXTL* (Sheldrick, 2008 ▶); software used to prepare material for publication: *SHELXTL*.

## Supplementary Material

Crystal structure: contains datablocks I, global. DOI: 10.1107/S1600536809038161/bt5068sup1.cif

Structure factors: contains datablocks I. DOI: 10.1107/S1600536809038161/bt5068Isup2.hkl

Additional supplementary materials:  crystallographic information; 3D view; checkCIF report

## Figures and Tables

**Table:** 

Te—C1	2.1242 (18)
Te—C10	2.136 (2)
Te—N	2.8079 (16)

**Table:** 

C1—Te—C10	94.19 (7)
C1—Te—N	70.77 (6)
C10—Te—N	164.92 (6)

**Fig. 1.**
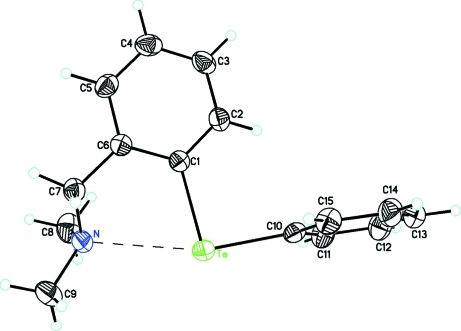
The molecular structure of C15H17NTe the showing the atom numbering scheme and 50% probability displacement ellipsoids. The secondary interaction between the N and Te is shown as a dashed line.

**Fig. 2.**
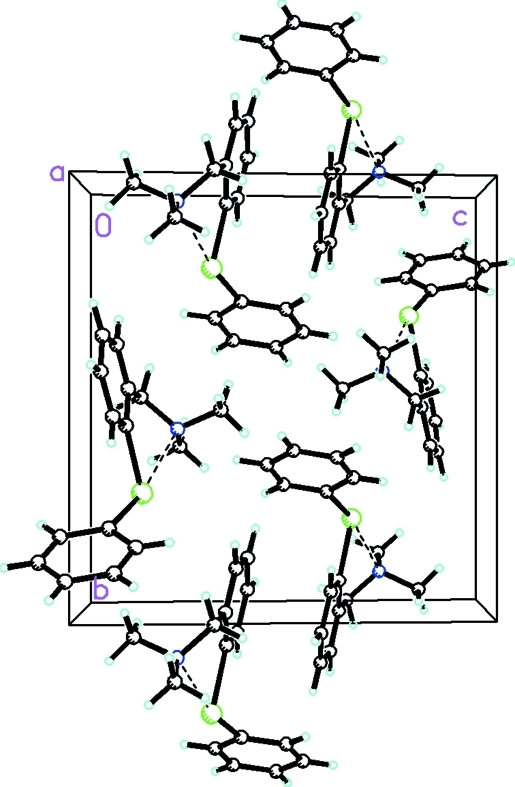
The molecular packing for C15H17NTe viewed down the a axis. The secondary interaction between the N and Te is shown by dashed lines.

## supplementary crystallographic information

### Comment

The stucture of the title compound, (I), is shown below. Dimensions are available in the archived CIF.

Hydrid Te, N ligands with soft Te and hard N are of current interest due to their intramolecular coordination as the *trans* bond is activated and easily cleaved by metals (Kaur *et al.* 2009). Thus incorporation of soft tellurium and hard nitrogen into mixed donor Te, N bidentate ligands makes them interesting and promising as candidates for catalysts in combination with soft transition metals like Pd and the Rh group metals. Their coordination properties can be varied by changing the nitrogen function. Thus by introduction of a secondary bonding interaction which weakens the bond *trans* to Te in *ortho*-coordinated or suitably arranged substrates, the catalytic transformation of tellurides as substrates in Heck-type (Nishibayashi *et al.* 1996*a*; Nishibayashi *et al.* 1996*b*) and cross coupling (Zeni & Comasseto, 1999; Zeni *et al.* 2001; Cella *et al.* 2006) reactions and their coordination properties (Kaur *et al.* 2009) can be influenced. Our group (Singh *et al.* 1990; Kaur *et al.* 1995; Menon *et al.* 1996; Panda *et al.* 1999) as well as others (Detty *et al.* 1995; Drake *et al.* 2001, Engman *et al.* 2004) have been involved in the synthesis and studies of such intramolecularly coordinated organotellurides. The title compound, but not the structure, has been reported previously by Detty and co-workers as well as by our group (Kaur *et al.* 1995).

In the structure of the title compound, considering the bonding geometry around the Te as V-shaped with a longer intramolecular Te···N secondary interaction, a pseudo five-membered puckered ring can be envisioned. The Te···N distance of 2.8079 (16) Å is much greater than the sum of their covalent radii (2.11 Å; Cordero *et al.* 2008) but less than the sum of their van der Waal radii (3.61 Å; Bondi, 1964) and greater than the corresponding distance in similar compounds *viz*., 2-NMe_2_CH_2_C_6_H_4_TeCl (2.362 (3) Å; Engman *et al.* 2004), 2-NMe_2_CH_2_C_6_H_4_TeI (2.366 (4) Å; Kaur *et al.* 1995) or 8-(dimethylamino)-1-naphthyl phenyl telluride (2.713 (1) Å; Menon *et al.* 1996). Due to this hypervalent intramolecular Te···N contact the Te—C bond (2.137 (2) Å) *trans* to the amino group gets slightly elongated compared to the other Te—C bond (2.1249 (18) Å). The bond angle for the *trans* N—Te—C atoms (164.92 (6)°) deviates significantly from linearity.

### Experimental

The title compound was prepared by the reported procedure (Kaur *et al.* 1995). A saturated solution of the compound was made in warm n-pentane and allowed to evaporate slowly at room temperature to grow crystals suitable for diffraction.

### Refinement

H atoms were placed in geometrically idealized positions and constrained to ride on their parent atoms with C—H distances ranging from 0.95 to 0.99 Å and *U*_iso_(H) = 1.2*U*_eq_(C) [1.5*U*_eq_(C) for CH_3_ H atoms].

### Crystal data

**Table:**

C_15_H_17_NTe	*F*(000) = 664
*M**_r_* = 338.90	*D*_x_ = 1.572 Mg m^−^^3^
Monoclinic, *P*2_1_/*c*	Mo *K*α radiation, λ = 0.71073 Å
Hall symbol: -P 2ybc	Cell parameters from 8619 reflections
*a* = 8.5736 (3) Å	θ = 4.9–32.4°
*b* = 13.2472 (5) Å	µ = 2.06 mm^−^^1^
*c* = 12.6719 (4) Å	*T* = 110 K
β = 95.933 (3)°	Prism, colorless
*V* = 1431.52 (9) Å^3^	0.49 × 0.41 × 0.27 mm
*Z* = 4	

### Data collection

**Table:**

Oxford Diffraction Gemini R CCD diffractometer	4836 independent reflections
Radiation source: Enhance (Mo) X-ray Source	2926 reflections with *I* > 2σ(*I*)
graphite	*R*_int_ = 0.028
Detector resolution: 10.5081 pixels mm^-1^	θ_max_ = 32.4°, θ_min_ = 4.9°
ω scans	*h* = −12→12
Absorption correction: multi-scan (*CrysAlis PRO*; Oxford Diffraction, 2009)	*k* = −18→19
*T*_min_ = 0.728, *T*_max_ = 1.000	*l* = −18→18
20642 measured reflections	

### Refinement

**Table:**

Refinement on *F*^2^	Primary atom site location: structure-invariant direct methods
Least-squares matrix: full	Secondary atom site location: difference Fourier map
*R*[*F*^2^ > 2σ(*F*^2^)] = 0.023	Hydrogen site location: inferred from neighbouring sites
*wR*(*F*^2^) = 0.058	H-atom parameters constrained
*S* = 0.97	*w* = 1/[σ^2^(*F*_o_^2^) + (0.0289*P*)^2^] where *P* = (*F*_o_^2^ + 2*F*_c_^2^)/3
4836 reflections	(Δ/σ)_max_ = 0.001
156 parameters	Δρ_max_ = 0.57 e Å^−^^3^
0 restraints	Δρ_min_ = −0.47 e Å^−^^3^

### Special details

**Table:**

Experimental. CrysAlis RED, (Oxford Diffraction, 2009) Empirical absorption correction using spherical harmonics, implemented in SCALE3 ABSPACK scaling algorithm.
Geometry. All e.s.d.'s (except the e.s.d. in the dihedral angle between two l.s. planes) are estimated using the full covariance matrix. The cell e.s.d.'s are taken into account individually in the estimation of e.s.d.'s in distances, angles and torsion angles; correlations between e.s.d.'s in cell parameters are only used when they are defined by crystal symmetry. An approximate (isotropic) treatment of cell e.s.d.'s is used for estimating e.s.d.'s involving l.s. planes.
Refinement. Refinement of *F*^2^ against ALL reflections. The weighted *R*-factor *wR* and goodness of fit *S* are based on *F*^2^, conventional *R*-factors *R* are based on *F*, with *F* set to zero for negative *F*^2^. The threshold expression of *F*^2^ > σ(*F*^2^) is used only for calculating *R*-factors(gt) *etc*. and is not relevant to the choice of reflections for refinement. *R*-factors based on *F*^2^ are statistically about twice as large as those based on *F*, and *R*- factors based on ALL data will be even larger.

### Fractional atomic coordinates and isotropic or equivalent isotropic displacement parameters (Å^2^)

**Table:**

	*x*	*y*	*z*	*U*_iso_*/*U*_eq_	
Te	0.886656 (14)	0.206826 (9)	0.327530 (10)	0.04605 (6)	
C7	1.0873 (2)	−0.00329 (16)	0.33777 (15)	0.0462 (5)	
H7A	1.1484	0.0230	0.4025	0.055*	
H7B	1.1349	−0.0681	0.3190	0.055*	
C1	0.8142 (2)	0.05794 (13)	0.36112 (13)	0.0373 (4)	
C2	0.6624 (2)	0.03853 (16)	0.38466 (14)	0.0460 (4)	
H2A	0.5888	0.0922	0.3840	0.055*	
C3	0.6175 (2)	−0.05790 (16)	0.40897 (16)	0.0533 (5)	
H3A	0.5134	−0.0704	0.4249	0.064*	
C4	0.7225 (3)	−0.13543 (17)	0.41016 (16)	0.0566 (5)	
H4A	0.6923	−0.2016	0.4287	0.068*	
C5	0.8725 (3)	−0.11769 (15)	0.38443 (15)	0.0497 (5)	
H5A	0.9435	−0.1726	0.3831	0.060*	
C6	0.9219 (2)	−0.02150 (14)	0.36041 (14)	0.0395 (4)	
N	1.09600 (18)	0.06793 (12)	0.25179 (13)	0.0470 (4)	
C8	1.0372 (3)	0.0248 (2)	0.14988 (17)	0.0711 (7)	
H8A	0.9302	−0.0002	0.1533	0.107*	
H8B	1.1050	−0.0311	0.1329	0.107*	
H8C	1.0366	0.0767	0.0947	0.107*	
C9	1.2542 (3)	0.1090 (2)	0.2514 (2)	0.0739 (7)	
H9A	1.2852	0.1426	0.3192	0.111*	
H9B	1.2559	0.1579	0.1934	0.111*	
H9C	1.3277	0.0540	0.2412	0.111*	
C10	0.6957 (2)	0.27717 (13)	0.39468 (15)	0.0433 (4)	
C11	0.5679 (3)	0.31290 (16)	0.32958 (17)	0.0554 (5)	
H11A	0.5633	0.3032	0.2550	0.066*	
C12	0.4467 (3)	0.36259 (18)	0.37197 (19)	0.0631 (6)	
H12A	0.3587	0.3856	0.3266	0.076*	
C13	0.4534 (3)	0.37856 (17)	0.47833 (19)	0.0609 (6)	
H13A	0.3709	0.4136	0.5071	0.073*	
C14	0.5789 (3)	0.34409 (18)	0.54402 (18)	0.0627 (6)	
H14A	0.5827	0.3548	0.6184	0.075*	
C15	0.6989 (3)	0.29428 (15)	0.50326 (16)	0.0536 (5)	
H15B	0.7856	0.2710	0.5497	0.064*	

### Atomic displacement parameters (Å^2^)

**Table:**

	*U*^11^	*U*^22^	*U*^33^	*U*^12^	*U*^13^	*U*^23^
Te	0.04845 (9)	0.04144 (8)	0.04904 (9)	0.00098 (6)	0.00871 (6)	0.00703 (6)
C7	0.0416 (10)	0.0500 (12)	0.0469 (11)	0.0059 (9)	0.0049 (8)	0.0024 (8)
C1	0.0395 (9)	0.0410 (10)	0.0314 (8)	−0.0007 (8)	0.0031 (7)	−0.0031 (7)
C2	0.0416 (10)	0.0501 (12)	0.0468 (10)	0.0001 (9)	0.0069 (8)	−0.0066 (9)
C3	0.0495 (11)	0.0563 (14)	0.0564 (12)	−0.0160 (10)	0.0169 (9)	−0.0145 (10)
C4	0.0731 (15)	0.0465 (13)	0.0521 (12)	−0.0174 (11)	0.0154 (10)	−0.0081 (9)
C5	0.0640 (13)	0.0393 (11)	0.0463 (11)	0.0035 (10)	0.0080 (9)	−0.0023 (9)
C6	0.0424 (10)	0.0416 (10)	0.0341 (9)	0.0011 (8)	0.0025 (7)	0.0013 (8)
N	0.0431 (9)	0.0491 (10)	0.0503 (9)	0.0027 (7)	0.0128 (7)	0.0049 (8)
C8	0.0833 (16)	0.0851 (19)	0.0466 (13)	0.0158 (15)	0.0149 (11)	0.0025 (12)
C9	0.0555 (13)	0.0756 (18)	0.0948 (18)	−0.0037 (12)	0.0279 (12)	0.0159 (14)
C10	0.0504 (11)	0.0327 (10)	0.0469 (11)	−0.0005 (8)	0.0049 (9)	0.0028 (8)
C11	0.0605 (13)	0.0542 (14)	0.0499 (12)	0.0097 (10)	−0.0022 (10)	−0.0012 (9)
C12	0.0563 (13)	0.0605 (15)	0.0712 (15)	0.0165 (11)	0.0005 (11)	0.0013 (12)
C13	0.0652 (14)	0.0455 (12)	0.0755 (15)	0.0070 (11)	0.0238 (12)	0.0006 (11)
C14	0.0827 (16)	0.0591 (15)	0.0486 (12)	0.0050 (12)	0.0171 (12)	−0.0042 (10)
C15	0.0603 (13)	0.0534 (13)	0.0462 (11)	0.0050 (10)	0.0006 (9)	0.0030 (9)

### Geometric parameters (Å, °)

**Table:**

Te—C1	2.1242 (18)	N—C9	1.462 (3)
Te—C10	2.136 (2)	C8—H8A	0.9800
Te—N	2.8079 (16)	C8—H8B	0.9800
C7—N	1.449 (2)	C8—H8C	0.9800
C7—C6	1.495 (2)	C9—H9A	0.9800
C7—H7A	0.9900	C9—H9B	0.9800
C7—H7B	0.9900	C9—H9C	0.9800
C1—C2	1.389 (3)	C10—C11	1.385 (3)
C1—C6	1.401 (3)	C10—C15	1.392 (3)
C2—C3	1.378 (3)	C11—C12	1.384 (3)
C2—H2A	0.9500	C11—H11A	0.9500
C3—C4	1.365 (3)	C12—C13	1.360 (3)
C3—H3A	0.9500	C12—H12A	0.9500
C4—C5	1.379 (3)	C13—C14	1.369 (3)
C4—H4A	0.9500	C13—H13A	0.9500
C5—C6	1.387 (3)	C14—C15	1.368 (3)
C5—H5A	0.9500	C14—H14A	0.9500
N—C8	1.454 (3)	C15—H15B	0.9500
			
C1—Te—C10	94.19 (7)	C9—N—Te	112.55 (14)
C1—Te—N	70.77 (6)	N—C8—H8A	109.5
C10—Te—N	164.92 (6)	N—C8—H8B	109.5
N—C7—C6	111.92 (14)	H8A—C8—H8B	109.5
N—C7—H7A	109.2	N—C8—H8C	109.5
C6—C7—H7A	109.2	H8A—C8—H8C	109.5
N—C7—H7B	109.2	H8B—C8—H8C	109.5
C6—C7—H7B	109.2	N—C9—H9A	109.5
H7A—C7—H7B	107.9	N—C9—H9B	109.5
C2—C1—C6	119.70 (17)	H9A—C9—H9B	109.5
C2—C1—Te	120.99 (14)	N—C9—H9C	109.5
C6—C1—Te	119.30 (13)	H9A—C9—H9C	109.5
C3—C2—C1	120.59 (18)	H9B—C9—H9C	109.5
C3—C2—H2A	119.7	C11—C10—C15	117.82 (19)
C1—C2—H2A	119.7	C11—C10—Te	120.25 (15)
C4—C3—C2	120.08 (19)	C15—C10—Te	121.81 (14)
C4—C3—H3A	120.0	C12—C11—C10	120.7 (2)
C2—C3—H3A	120.0	C12—C11—H11A	119.6
C3—C4—C5	119.9 (2)	C10—C11—H11A	119.6
C3—C4—H4A	120.0	C13—C12—C11	120.2 (2)
C5—C4—H4A	120.0	C13—C12—H12A	119.9
C4—C5—C6	121.48 (19)	C11—C12—H12A	119.9
C4—C5—H5A	119.3	C12—C13—C14	120.0 (2)
C6—C5—H5A	119.3	C12—C13—H13A	120.0
C5—C6—C1	118.17 (17)	C14—C13—H13A	120.0
C5—C6—C7	120.53 (17)	C15—C14—C13	120.4 (2)
C1—C6—C7	121.27 (17)	C15—C14—H14A	119.8
C7—N—C8	111.84 (17)	C13—C14—H14A	119.8
C7—N—C9	111.41 (16)	C14—C15—C10	120.87 (19)
C8—N—C9	112.33 (17)	C14—C15—H15B	119.6
C7—N—Te	94.88 (10)	C10—C15—H15B	119.6
C8—N—Te	112.69 (13)		
			
C10—Te—C1—C2	17.26 (15)	C6—C7—N—Te	45.84 (15)
N—Te—C1—C2	−161.59 (15)	C1—Te—N—C7	−35.48 (10)
C10—Te—C1—C6	−162.12 (13)	C10—Te—N—C7	−39.9 (3)
N—Te—C1—C6	19.02 (12)	C1—Te—N—C8	80.65 (15)
C6—C1—C2—C3	1.0 (3)	C10—Te—N—C8	76.3 (3)
Te—C1—C2—C3	−178.36 (13)	C1—Te—N—C9	−151.08 (15)
C1—C2—C3—C4	0.0 (3)	C10—Te—N—C9	−155.5 (2)
C2—C3—C4—C5	−1.6 (3)	C1—Te—C10—C11	−101.37 (16)
C3—C4—C5—C6	2.2 (3)	N—Te—C10—C11	−97.2 (3)
C4—C5—C6—C1	−1.1 (3)	C1—Te—C10—C15	82.60 (16)
C4—C5—C6—C7	177.09 (17)	N—Te—C10—C15	86.8 (3)
C2—C1—C6—C5	−0.5 (3)	C15—C10—C11—C12	−0.9 (3)
Te—C1—C6—C5	178.88 (13)	Te—C10—C11—C12	−177.11 (17)
C2—C1—C6—C7	−178.66 (16)	C10—C11—C12—C13	1.2 (4)
Te—C1—C6—C7	0.7 (2)	C11—C12—C13—C14	−1.0 (4)
N—C7—C6—C5	140.26 (18)	C12—C13—C14—C15	0.6 (4)
N—C7—C6—C1	−41.6 (2)	C13—C14—C15—C10	−0.3 (4)
C6—C7—N—C8	−71.0 (2)	C11—C10—C15—C14	0.5 (3)
C6—C7—N—C9	162.38 (17)	Te—C10—C15—C14	176.60 (17)
